# *Blastocystis* prevalence and subtypes in autochthonous and immigrant patients in a referral centre for parasitic infections in Italy

**DOI:** 10.1371/journal.pone.0210171

**Published:** 2019-01-07

**Authors:** Chiara Piubelli, Hossein Soleymanpoor, Giovanni Giorli, Fabio Formenti, Dora Buonfrate, Zeno Bisoffi, Francesca Perandin

**Affiliations:** 1 Department of Infectious and Tropical Diseases, IRCCS Sacro Cuore Don Calabria Hospital, Negrar (Vr), Italy; 2 Institute of Infectious Diseases and Public Health, Università Politecnica delle Marche, Ancona, Italy; Charles University, CZECH REPUBLIC

## Abstract

In this study we characterized the presence and subtype (ST1-ST4) of *Blastocystis* in patients attended at a referral center for tropical diseases in Northern Italy. We also, evaluated the organism’s association with other intestinal parasites. Parasite screening was performed on 756 patients, from different geographical origins (namely, Italians, Africans, South Americans, Asian and non-Italian Europeans) in which Italians represented the largest group. *Blastocystis* was seen to be the most prevalent parasite in the study. Subtype 3 and 1 were the most frequently found in the Italians and Africans. Our data confirmed previous studies performed in Italy, in which ST3 proved to be the most prevalent subtype, but we highlighted also a high frequency of mixed subtypes, which were probably underestimated in former analyses. Interestingly, the mixed subtypes group was the most prevalent in all the analysed geographical areas. About half of our cases showed other co-infecting parasites and the most frequent was *Dientamoeba fragilis*. Our study confirms that, in *Blastocystis* infection, multiple subtypes and co-infecting parasites are very frequently present, in particular *Dientamoeba fragilis*.

## Introduction

*Blastocystis* is a common enteric protist, belonging to the heterogeneous infrakindom of Stramenopiles [1]. It has a worldwide distribution and is transmitted by fecal-oral direct contact or waterborne transmission [2, 3]. The pathogenicity of the parasite remains controversial. Several publications suggest an association with gastrointestinal (GI) symptoms such as diarrhea, abdominal pain, nausea, vomiting, fatigue, flatulence, irritable bowel syndrome (IBS) and inflammatory bowel disease (IBD) [4–8]. In the majority of these cases, GI symptoms can be successfully treated with antiparasitic drugs [5, 6]. Moreover, an association with cutaneous symptoms and urticaria is also suggested [9, 10]. Nevertheless, there are also a number of studies in which *Blastocystis* spp, is found in feces from many asymptomatic individuals [11–13]. Recent molecular genetic studies demonstrated the existence of ten subtypes (STs) in humans (ST1-ST9 and ST12[5, 13]), classified according to different small-subunit (SSU) ribosomal lineages [14]. Subtype determination can be obtained by the molecular analysis of a particular genomic region of the SSU-ribosomal DNA (rDNA) [15–17]. Whether pathogenicity is linked to a particular subtype or association with other parasite co-infections remains unclear. Moreover, the incidence of mixed-ST infections was often not considered or underestimated, due to methodological issues [17].

Different studies demonstrated that subtypes 1 to 4 are the most common in humans [5, 6, 18] and that they have different geographical distributions.

In the present study, fecal specimens collected from patients suspected of harboring intestinal parasites and attending our center for tropical disease between 2014 and 2015 were screened for the presence of *Blastocystis* DNA. The aim of the study was to characterize the distribution of *Blastocystis* subtypes in our patients, evaluating: *i*) the influence of the geographical origin, *ii*) the dynamics of mixed STs and *iii*) the association with other parasite co-infections, in order to explore the prevalence and the diversity of *Blastocysti*s infections in our cohort population. In order to highlight the presence of mixed-ST infections, we applied the quick and sensitive nested-PCR method described by Scanlan et al [17] and developed for large studies with multiple samples, focusing the analysis on the four most frequent STs (ST1-ST4). This method is reported to be the most sensitive in the detection of mixed infections, circumventing laborious clone library preparations [17].

## Materials and methods

### Setting and participants

This retrospective study was performed at the Centre for Tropical Diseases of the IRCCS Sacro Cuore Don Calabria Hospital in Negrar (Verona), a referral center for tropical medicine in Veneto region. Patients attending our center are mostly Italians mainly coming from Veneto and neighboring regions, missionaries who need medical assistance, and recently arrived immigrants. According to our clinical protocols, we carry out the first-level molecular parasitology screening in patients with gastrointestinal symptoms and/or eosinophilia, and in immigrants and adopted children coming from Africa, Asia and South America, irrespective of symptoms/signs.

The sample identification code was retrieved from the electronic database of the molecular parasitology laboratory, searching among all fecal specimens collected from January 2014 to December 2015 and submitted to molecular screening for intestinal parasites. The patients’ age ranged from 0 to 88 years. Although patients are usually referred to our center for intestinal parasites investigation after the exclusion of other infections, we could not exclude the presence of potential viral or bacterial pathogens, since this information was not available for all the samples.

### Ethics approval and consent to participate

Fecal samples were collected in accordance with the requirements of the Declaration of Helsinki; all patients included in this study gave their written consent to the donation of their biological samples for research purpose. All data were fully anonymized before the retrospective analysis. The study protocol received ethical clearance by the local competent Ethics Committee (Comitato Etico per la Sperimentazione Clinica delle Province di Verona e Rovigo, protocol number 34680, 2017).

### Variables and study size

In the two-year period, 756 samples (one fecal specimen per each subject) were tested for the presence of *Blastocystis spp* by real-time polymerase chain reaction (Rt-PCR).

Samples positive for *Blastocystis spp*. were further characterized for the presence of the four most frequent human subtypes (ST1, ST2, ST3 and ST4) by a specific nested-PCR. The clinical records of patients submitted to *Blastocystis* subtypes analysis were searched in order to classify the cases in one of the following categories: presence of GI (IBS, IBD, abdominal pain, diarrhea) symptoms, presence of itching, no symptoms. Age, sex and geographical origin of the patients (Italians, non-Italian Europeans, Africans, Asians and South Americans) were considered. No information was available about occupational activity or travelling history. The presence of other possible parasites was also retrieved from the database of the molecular parasitology laboratory.

### DNA extraction and molecular screening for intestinal parasites

According to the routine procedure of our laboratory, Fecal samples collection and DNA extraction was performed according to the routine procedure of our laboratory, as previously described [19]. Molecular diagnostic screening for intestinal parasite was performed by three separate multiplex Rt-PCRs for *Entamoeba histolytica—Entamoeba dispar—Cryptosporidium spp*., for *Giardia intestinalis—Dientamoeba fragilis—Blastocystis spp*. and for *Strongyloides stercoralis—Schistosoma spp—Hymenolepis nana*. Multiplex Rt-PCRs were performed adapting the protocol reported by Verweij and colleagues [20, 21]. Details on methods are available in Protocols.io open access repository at the following link: dx.doi.org/10.17504/protocols.io.sf9ebr6.

### *Blastocystis* subtype analysis

Nested-PCR was adapted from Scanlan *et al*. [17]. Briefly, a first step PCR was performed to provide a *Blastocystis* specific 18S rDNA template for each of the subsequent ST-specific PCRs (ST1, ST2, ST3, and ST4). RD5, BhRDr, ST1-F, ST2-F, ST3-F, ST4-F primers sequences were retrieved from Scanlan *et al*. [17] and PCR was performed using iTaq DNA polymerase (Bio-Rad) in 50 μL of reaction volume, according to the manufacturer’s instructions. The following cycling conditions were applied for the first step PCR: initial denaturation 95 °C for 3 min, 30 cycles at 94 °C for 1 min, 59 °C for 1 min, 72 °C for 1 min, final elongation 72 °C for 5 min. 5 μL of DNA sample was used. The ST-specific PCRs were performed as follows: initial denaturation 95 °C for 3 min, 35 cycles at 94 °C for 30 sec, T_annealing_ primers for 30 sec, 72°C for 1 min, final elongation 72 °C for 5 min. The following T_annealing_ were used: 56 °C for ST1 and ST2, 48 °C for ST3 and ST4. 1 μL the initial PCR product was loaded per each reaction. A no-template control was always included in each PCR run. PCR products were analysed by 2.5% agarose gel electrophoresis, to detect the specific DNA bands [17]. An example of gel image is reported in supplementary figure (S1 Fig).

### Statistical analysis

Descriptive statistical analysis was carried out for the entire cohort and separately for each continent of origin of the patients. The categorical variables were reported as frequencies and proportions, while the quantitative variables were presented as means with standard deviations (SD).

We then investigated on associations between all patients’ characteristics through univariate logistic regression models, parametric and non-parametric statistical tests, such as Student’s T-Test and Chi-Squared test. All statistical analyses were conducted using R, version 3.3.3 [22].

## Results

The retrospective analysis of our molecular parasitology database retrieved a set of 509 subjects with intestinal parasite’s infections, of whom 258 were positive for *Blastocystis*. We found a higher prevalence of *Blastocystis* infection in males than in females (38.5% and 28.8% respectively). No association with age was observed (t-test, p = 0.264). *Blastocystsis* subtype analysis was successful for 221 samples out of 258. The characterized samples were stratified according to the geographical origin of the subjects. Details on subtype distribution across the different geographical groups, as well as the demographic characteristics of the subjects are reported in Table 1.

**Table 1:** Baseline demographic characteristics of the *Blastocystis* positive Cohort, stratified by area of origin.

Characteristic	Entire Cohort	Area of Origin^a^				
	N,%	Italy	Europe	Africa	South America	Asia
**Female. N (%)**	86 (38.9)	46 (49.5)	3 (42.9)	17 (22.4)	15 (60.0)	5 (25.0)
**Male. N (%)**	135 (61.1)	47 (50.5)	4 (57.1)	59 (77.6)	10 (40.0)	15 (75.0)
**Total**	221	93 (42.1)	7 (3.2)	76 (34.4)	25 (11.3)	20 (9.0)
**Mean age, years (SD)**	35.9 (19.6)	48.6 (17.3)	22.4 (13.5)	25.8 (13.7)	37.3 (20.3)	18.9 (10.6)
***Blastocystis* Subtype**^b^, **N (%)**						
**Subtype 1**	51 (23.2)	20 (21.5)	1 (14.2)	20 (26.3)	4 (16.0)	6 (30.0)
**Subtype 2**	17 (7.7)	10 (10.7)	2 (28.6)	4 (5.3)	1 (4.0)	0 (0.0)
**Subtype 3**	59 (26.7)	22 (23.7)	2 (28.6)	20 (26.3)	9 (36.0)	6 (30.0)
**Subtype 4**	14 (6.3)	9 (9.7)	0 (0.0)	4 (5.3)	1 (4.0)	0 (0.0)
**Combination**	80 (36.1)	32 (34.4)	2 (28.6)	28 (36.8)	10 (40.0)	8 (40.0)

Abbreviations: SD, standard deviation; GI, gastrointestinal.

^a^ European patients are coming from European countries other than Italy, namely: Germany, Romania, Switzerland and European Russia.

^b^ We here report the presence of a single subtype. Combination indicates mixed-ST infections that include any possible combination of the above.

ST3 and ST1 were the most frequent subtypes in our cohort, with a prevalence of 26.7% and 23.1%, respectively (Table 1). Focusing on the two most representative areas of origin, ST1 and ST3 were confirmed as the predominant subtypes in both Italians and Africans. In all the analyzed geographical areas, the most prevalent group was composed by subjects infected by more than one *Blastocystis* subtype.

**Fig 1:**
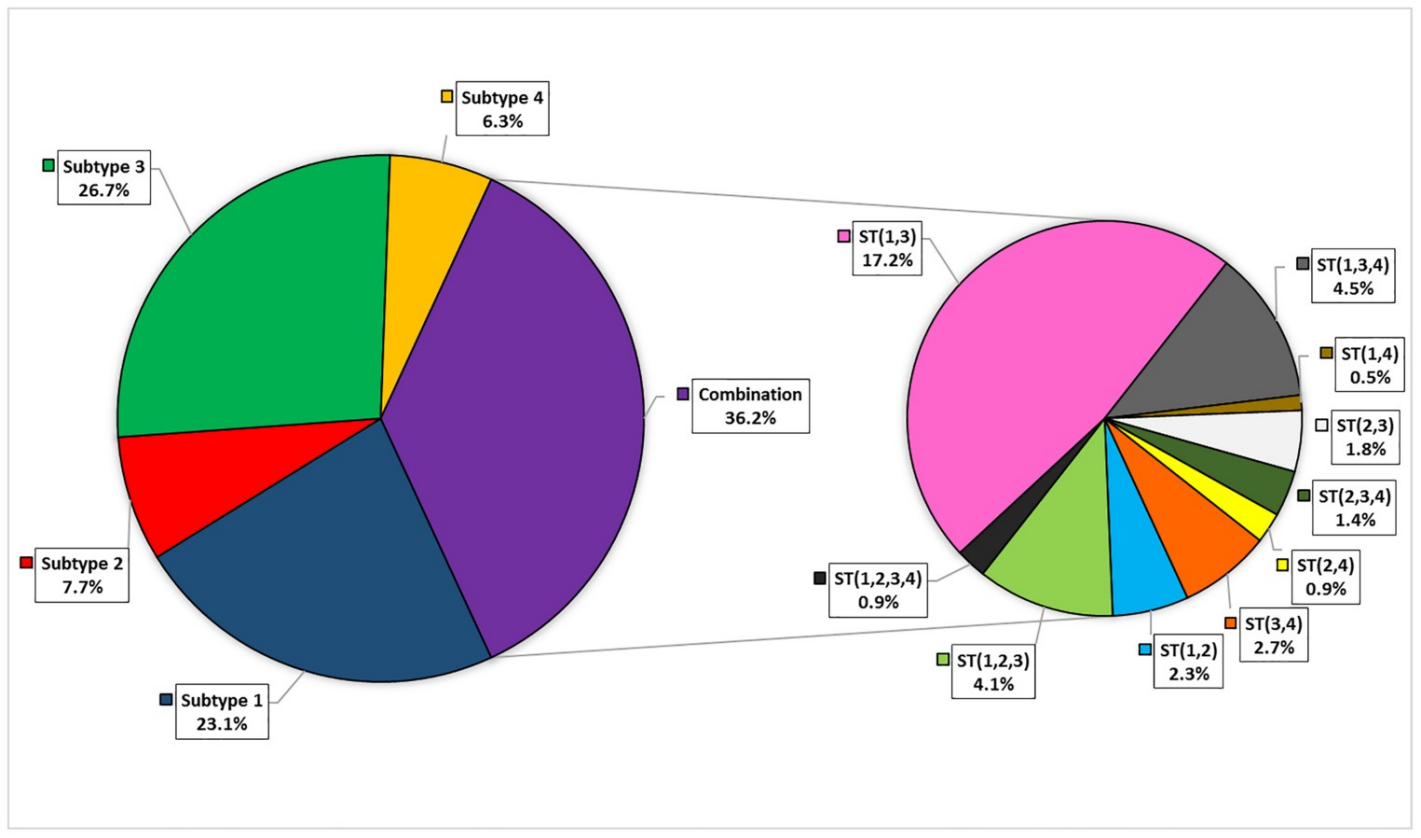
*Blastocystis* subtype distribution. The left pie indicates the presence of a single subtype; the pie on the right represents a zoom on the detected different combinations of mixed subtypes.

The most frequent mixed-ST was clearly the ST1-ST3 (Fig 1), for almost all the analyzed geographical areas (Table 2). Although at a lower frequency, triple STs infections were also detected, for instance ST1-ST2-ST3 and ST1-ST3-ST4.

**Table 2:** *Blastocystis* mixed-ST distribution according to the area of origin.

Area of origin	ST1-ST3	ST1-ST3-ST4	ST1-ST2-ST3	ST3-ST4	Others
**Italy**	16(42.1)	5(50)	2(22.2)	2(33.3)	7(41.2)
**Europe**	0(0)	1(10)	0(0)	0(0)	1(5.9)
**Africa**	13(34.2)	3(30)	2(22.2)	1(16.7)	9(52.9)
**South America**	5(13.2)	0(0)	3(33.3)	2(33.3)	0(0)
**Asia**	4(10.5)	1(10)	2(22.2)	1(16.7)	0(0)
**TOT**	38(100)	10(100)	9(100)	6(100)	17(100)

ST1-ST3, ST1-ST3-ST4, ST1-ST2-ST3, ST3-ST4 indicate double or triple subtypes infections; Others indicate all the other unmentioned subtypes combinations.

We evaluated a possible correlation between a particular subtype and age or sex, in any geographical region, but we did not find any significant association (data not reported).

**Fig 2:**
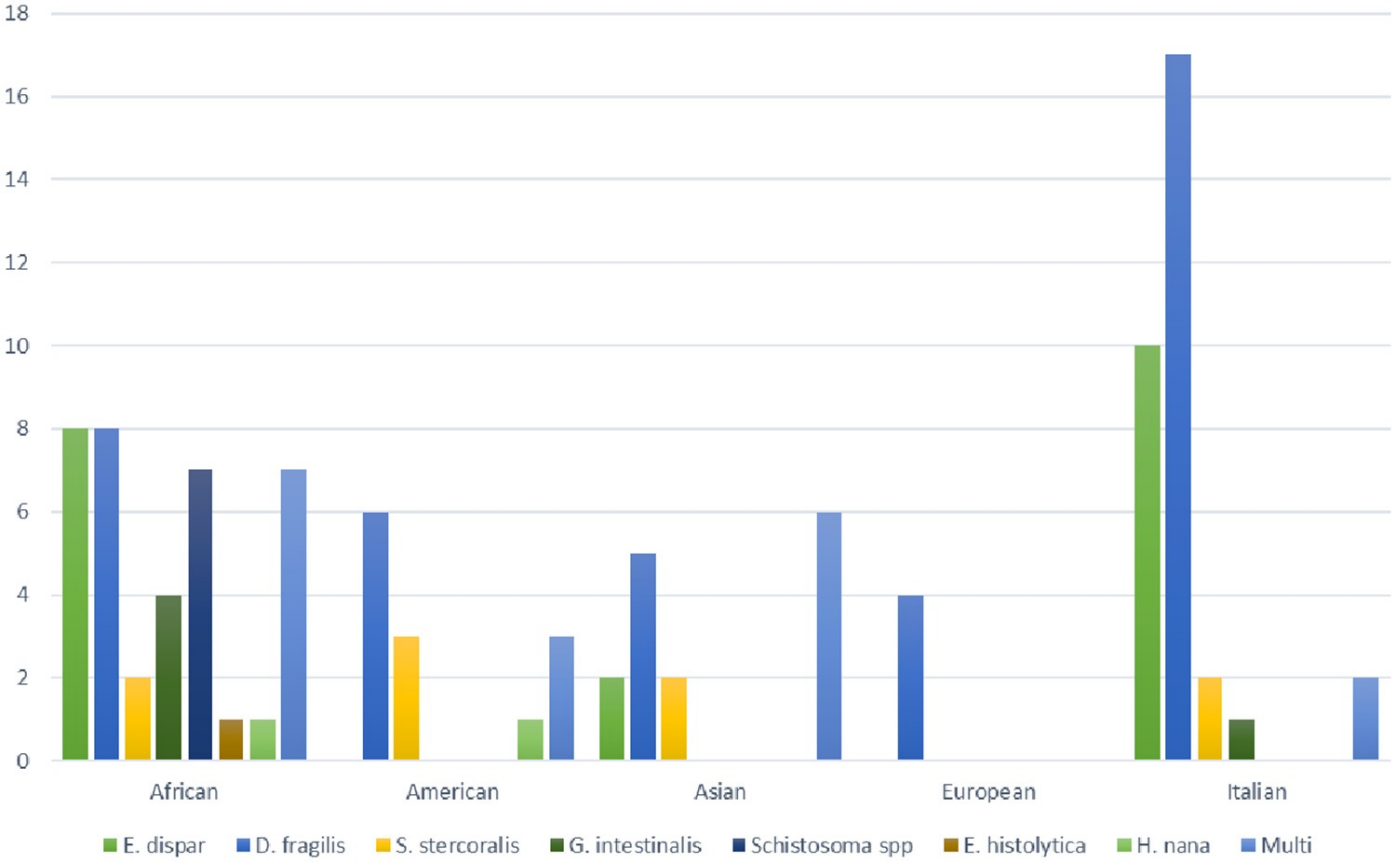
Co-infecting parasites. The bar chart represents the different parasites co-infections in *Blastocystis* positive subjects, across the available geographical areas.

Analysis of co-infecting parasites revealed that 46% of *Blastocystis*-positive samples had co-infections. No association was detected between a particular *Blastocystis* subtype and other parasites (data not reported), while we observed an inverse correlation between age and the occurrence of other co-infections (average ages: 31.7 *vs* 39.6 years of co-infected and non-co-infected patients, respectively; p = 0.004). Fig 2 shows the different co-infecting parasites detected across the geographical groups in our cohort.

*Dientamoeba fragilis* (*D*. *fragilis*) was the most frequent co-infecting parasite in all the geographical areas (Fig 2), with 24% (53/221) of analyzed cases harboring also this parasite.

Considering this result, we further explored the possible correlation between *Blastocystis* and *D*. *fragilis*, finding a statistically significant association between the two parasites (OR: 1.86, 95% CI: 1.27 to 2.73). We also found an inversely proportional association between *D*. *fragilis* infection and age (OR: 0.97, 95% CI: 0.96 to 0.98).

## Discussion

*Blastocystis* emerged as the most frequent intestinal parasite in patients referring to our Centre for Tropical Diseases, confirming previous reports indicating that *Blastocystis* infection has a higher prevalence than other intestinal parasites [23]. We could characterize *Blastocystis* subtype for 221 out of 258 positive samples. The presence of 37 uncharacterized samples was due to the applied method, which detects only the 4 most frequent subtypes (ST1, ST2, ST3 and ST4) and/ or to the low amount of available DNA. In fact, for 20 samples the DNA quantity was insufficient to complete the ST analysis, while 17 samples gave negative results. Among these 17 samples, 9 were from Italian, 6 from African, 1 from South American and 1 from European subjects. All the 17 samples gave very positive signals at the *Blastocystis spp* test (17.33≤Ct≤32.5 by real-time PCR and a detectable signal in the first step of the nested PCR), but they were negative at the ST1-4 specific test. The latter data suggest the presence of a ST different from ST1-ST4 in the 6.6% of subjects in our cohort. Several studies demonstrated that subtypes 1 to 4 are the most common in humans [5, 6, 18] and that they have different geographical distributions. Two previous studies confirmed that ST1-ST4 are predominant also in the Italian population [24, 25], even though 4% of individuals presented different subtypes [in a total of 223 subjects, six carried ST6 [25], one ST7 [24] and two ST8 [24, 25]]. Both these studies applied the method of 18S rDNA barcoding [15], using universal primers and sequencing. As indicated by Stensvold and Clark in 2016 [5], the drawbacks of barcoding is that mixed subtype infections may not always be evident in sequence chromatograms and can be underestimated. In these studies, only for a small number of samples the PCR products were cloned and sequenced in duplicates, thus revealing the presence of four mixed-ST infections out of 34 cloned samples [24]. Compared to the two previous Italian studies referred above, an intriguing result of our analysis was the frequent presence of mixed subtype infections, across all the geographical areas. This observation points out how a sensitive subtype-specific PCR assay could highlight a frequent presence of mixed subtype co-infections [17]. This aspect received little attention in the past, since it was often underestimated by methodological limits and considered just an incidental finding. Recently, the presence of mixed subtype infections was demonstrated to be an important characteristic, in order to explore the diversity and distribution of this parasite in the human gut [17]. Applying the subtype-specific method updated by Scanlan et al. [17], we were able to detect mixed infections in 36.1% of our cases, with ST1-ST3 being the most common mixed subtype combination, thus confirming previous reports [7].

In our cohort, we had the opportunity to compare subjects coming from different geographical areas. Our analysis highlighted that ST3 was the most prevalent subtype in Italians (23.7%), and ST1 was present at a slightly lower frequency (21.5%). ST3 was reported to be the most human-specific subtype [23, 26], thus suggesting a human-to-human transmission. The two previous studies conducted in Italy [24, 25] observed a higher frequency of ST3 (about 45%) and a lower frequency of ST1 (about 15%) in Italian patients. This could be due to the different regional origin of the patients’ population (our center is in northern Italy, while the other two studies were located in central Italy). In Africans, the second predominant geographical group in our cohort, ST1 and ST3 were the most prevalent subtypes, with equal frequency. Forsell and colleagues reviewed the subtype prevalence in Africa and, depending on the considered country, they reported ST1 and ST3 as actually being the most prevalent subtypes [27]. No particular subtype has been consistently linked to symptoms so far [28]. An association between *Blastocystis spp* and other parasites has already been observed [e.g. with *G*. *intestinalis* [27], and with *D*. *fragilis* [29]] but with no conclusive correlation with symptoms. The statistically significant association between *Blastocystis* and *D*. *fragilis* might indicate a cooperative interaction between the two protozoa. *D*. *fragilis* is also a commonly diagnosed parasite of the human gut and, as is the case with *Blastocystis*, its pathogenic role is still controversial [30].

The present study has some limitations, mainly due to the retrospective design. In particular, we could not accurately evaluate symptoms in our cohort. Furthermore, since the study has been conducted in patients suspected of harboring intestinal parasites, attending to our centre for tropical medicine, the results may not be representative of the general population. Moreover, we failed to characterize *Blastocystis* subtype in about 14% of the samples for the reasons outlined above.

## Conclusions

Our study population confirms a high prevalence of mixed-ST in all the different geographical groups. We found a significant association between *Blastocystis* and *D*. *fragilis* that might indicate a cooperative interaction between the two protozoa. Further prospective studies on the potential clinical relevance of *Blastocystis* subtypes should be designed, exploring also the patients’ immunological conditions and possible changes of gut microbiota associated to *Blastocystis* infection.

## Supporting information

S1 Fig Nested PCR for *Blastocystis* genotype detection on agarose gel. From left to right, lane 1 displays 50 bp DNA Step Ladder (Sigma), lane 2 is negative control, lane 3 displays first step PCR product (607 bp) and lines 6, 5, 4, and 7 display second step PCR products, respectively: ST1 (433 bp), ST2 (459 bp), ST3 (427 bp) and ST4 (399 bp).
(TIF)

S1 Table *Blastocystis* positive Cohort dataset. Fully anonymized data are reported for all the subjects included in the analysis.
(XLS)

## References

[1] Silberman JD, Sogin ML, Leipe DD, Clark CG. Human parasite finds taxonomic home. Nature. 1996;380(6573):398 10.1038/380398a0 . 8602239

[2] Lee IL, Tan TC, Tan PC, Nanthiney DR, Biraj MK, Surendra KM, et al Predominance of Blastocystis sp. subtype 4 in rural communities, Nepal. Parasitology research. 2012;110(4):1553–62. 10.1007/s00436-011-2665-0 . 22076050

[3] Li LH, Zhou XN, Du ZW, Wang XZ, Wang LB, Jiang JY, et al Molecular epidemiology of human Blastocystis in a village in Yunnan province, China. Parasitology international. 2007;56(4):281–6. 10.1016/j.parint.2007.06.001 . 17627869

[4] Roberts T, Stark D, Harkness J, Ellis J. Update on the pathogenic potential and treatment options for Blastocystis sp. Gut pathogens. 2014;6:17 10.1186/1757-4749-6-17 . 24883113 PMC4039988

[5] Stensvold CR, Clark CG. Current status of Blastocystis: A personal view. Parasitology international. 2016;65(6 Pt B):763–71. 10.1016/j.parint.2016.05.015 . 27247124

[6] Lepczynska M, Bialkowska J, Dzika E, Piskorz-Ogorek K, Korycinska J. Blastocystis: how do specific diets and human gut microbiota affect its development and pathogenicity? European journal of clinical microbiology & infectious diseases: official publication of the European Society of Clinical Microbiology. 2017 10.1007/s10096-017-2965-0 . 28326446 PMC5554277

[7] Sekar Uma S M. Recent insights into the genetic diversity, epidemiology and clinical relevance of Blastocystis species. The Journal of Medical Research 2015;1(1):33–9.

[8] Poirier P, Wawrzyniak I, Vivares CP, Delbac F, El Alaoui H. New insights into Blastocystis spp.: a potential link with irritable bowel syndrome. PLoS pathogens. 2012;8(3):e1002545 10.1371/journal.ppat.1002545 . 22438803 PMC3305450

[9] Lepczynska M, Chen WC, Dzika E. Mysterious chronic urticaria caused by Blastocystis spp.? International journal of dermatology. 2016;55(3):259–66; quiz 63–4, 66. 10.1111/ijd.13064 . 26469206

[10] Kolkhir P, Balakirski G, Merk HF, Olisova O, Maurer M. Chronic spontaneous urticaria and internal parasites—a systematic review. Allergy. 2016;71(3):308–22. 10.1111/all.12818 . 26648083

[11] Scanlan PD, Stensvold CR, Rajilic-Stojanovic M, Heilig HG, De Vos WM, O’Toole PW, et al The microbial eukaryote Blastocystis is a prevalent and diverse member of the healthy human gut microbiota. FEMS microbiology ecology. 2014;90(1):326–30. 10.1111/1574-6941.12396 . 25077936

[12] Kaneda Y, Horiki N, Cheng X, Tachibana H, Tsutsumi Y. Serologic response to Blastocystis hominis infection in asymptomatic individuals. The Tokai journal of experimental and clinical medicine. 2000;25(2):51–6. . 11127507

[13] Ramirez JD, Sanchez A, Hernandez C, Florez C, Bernal MC, Giraldo JC, et al Geographic distribution of human Blastocystis subtypes in South America. Infection, genetics and evolution: journal of molecular epidemiology and evolutionary genetics in infectious diseases. 2016;41:32–5. 10.1016/j.meegid.2016.03.017 . 27034056

[14] Stensvold CR, Suresh GK, Tan KS, Thompson RC, Traub RJ, Viscogliosi E, et al Terminology for Blastocystis subtypes—a consensus. Trends in parasitology. 2007;23(3):93–6. 10.1016/j.pt.2007.01.004 . 17241816

[15] Scicluna SM, Tawari B, Clark CG. DNA barcoding of blastocystis. Protist. 2006;157(1):77–85. 10.1016/j.protis.2005.12.001 . 16431158

[16] Stensvold CR. Comparison of sequencing (barcode region) and sequence-tagged-site PCR for Blastocystis subtyping. Journal of clinical microbiology. 2013;51(1):190–4. 10.1128/JCM.02541-12 . 23115257 PMC3536234

[17] Scanlan PD, Stensvold CR, Cotter PD. Development and Application of a Blastocystis Subtype-Specific PCR Assay Reveals that Mixed-Subtype Infections Are Common in a Healthy Human Population. Applied and environmental microbiology. 2015;81(12):4071–6. 10.1128/AEM.00520-15 . 25841010 PMC4524157

[18] Alfellani MA, Taner-Mulla D, Jacob AS, Imeede CA, Yoshikawa H, Stensvold CR, et al Genetic diversity of blastocystis in livestock and zoo animals. Protist. 2013;164(4):497–509. 10.1016/j.protis.2013.05.003 . 23770574

[19] Formenti F, Perandin F, Bonafini S, Degani M, Bisoffi Z. [Evaluation of the new ImmunoCard STAT!(R) CGE test for the diagnosis of Amebiasis]. Bulletin de la Societe de pathologie exotique. 2015;108(3):171–4. 10.1007/s13149-015-0434-5 . 26018388

[20] Verweij JJ, Mulder B, Poell B, van Middelkoop D, Brienen EA, van Lieshout L. Real-time PCR for the detection of Dientamoeba fragilis in fecal samples. Molecular and cellular probes. 2007;21(5–6):400–4. 10.1016/j.mcp.2007.05.006 . 17587544

[21] Verweij JJ, Oostvogel F, Brienen EA, Nang-Beifubah A, Ziem J, Polderman AM. Short communication: Prevalence of Entamoeba histolytica and Entamoeba dispar in northern Ghana. Tropical medicine & international health: TM & IH. 2003;8(12):1153–6. . 14641852 10.1046/j.1360-2276.2003.01145.x

[22] R-CoreTeam. R: A Language and Environment for Statistical Computing. R Foundation for Statistical Computing. 2017:http://www.R-project.org/.

[23] Tan KS. New insights on classification, identification, and clinical relevance of Blastocystis spp. Clinical microbiology reviews. 2008;21(4):639–65. 10.1128/CMR.00022-08 . 18854485 PMC2570156

[24] Meloni D, Sanciu G, Poirier P, El Alaoui H, Chabe M, Delhaes L, et al Molecular subtyping of Blastocystis sp. isolates from symptomatic patients in Italy. Parasitology research. 2011;109(3):613–9. 10.1007/s00436-011-2294-7 . 21340563

[25] Mattiucci S, Crisafi B, Gabrielli S, Paoletti M, Cancrini G. Molecular epidemiology and genetic diversity of Blastocystis infection in humans in Italy. Epidemiology and infection. 2016;144(3):635–46. . 26194649 10.1017/S0950268815001697

[26] Noel C, Dufernez F, Gerbod D, Edgcomb VP, Delgado-Viscogliosi P, Ho LC, et al Molecular phylogenies of Blastocystis isolates from different hosts: implications for genetic diversity, identification of species, and zoonosis. Journal of clinical microbiology. 2005;43(1):348–55. 10.1128/JCM.43.1.348-355.2005 . 15634993 PMC540115

[27] Forsell J, Granlund M, Samuelsson L, Koskiniemi S, Edebro H, Evengard B. High occurrence of Blastocystis sp. subtypes 1–3 and Giardia intestinalis assemblage B among patients in Zanzibar, Tanzania. Parasites & vectors. 2016;9(1):370 10.1186/s13071-016-1637-8 . 27356981 PMC4928263

[28] Clark CG, van der Giezen M, Alfellani MA, Stensvold CR. Recent developments in Blastocystis research. Advances in parasitology. 2013;82:1–32. 10.1016/B978-0-12-407706-5.00001-0 . 23548084

[29] Bart A, Wentink-Bonnema EM, Gilis H, Verhaar N, Wassenaar CJ, van Vugt M, et al Diagnosis and subtype analysis of Blastocystis sp. in 442 patients in a hospital setting in the Netherlands. BMC infectious diseases. 2013;13:389 10.1186/1471-2334-13-389 . 23972160 PMC3765316

[30] Caccio SM. Molecular epidemiology of Dientamoeba fragilis. Acta tropica. 2017 10.1016/j.actatropica.2017.06.029 . 28697994

