# Supplementary figures and images for: Blastocystis prevalence and subtypes in autochthonous and immigrant patients in a referral centre for parasitic infections in Italy
Source: PLoS One. 2019 Jan 7;14(1):e0210171. doi: 10.1371/journal.pone.0210171 (PMC6322732; doi:10.1371/journal.pone.0210171)

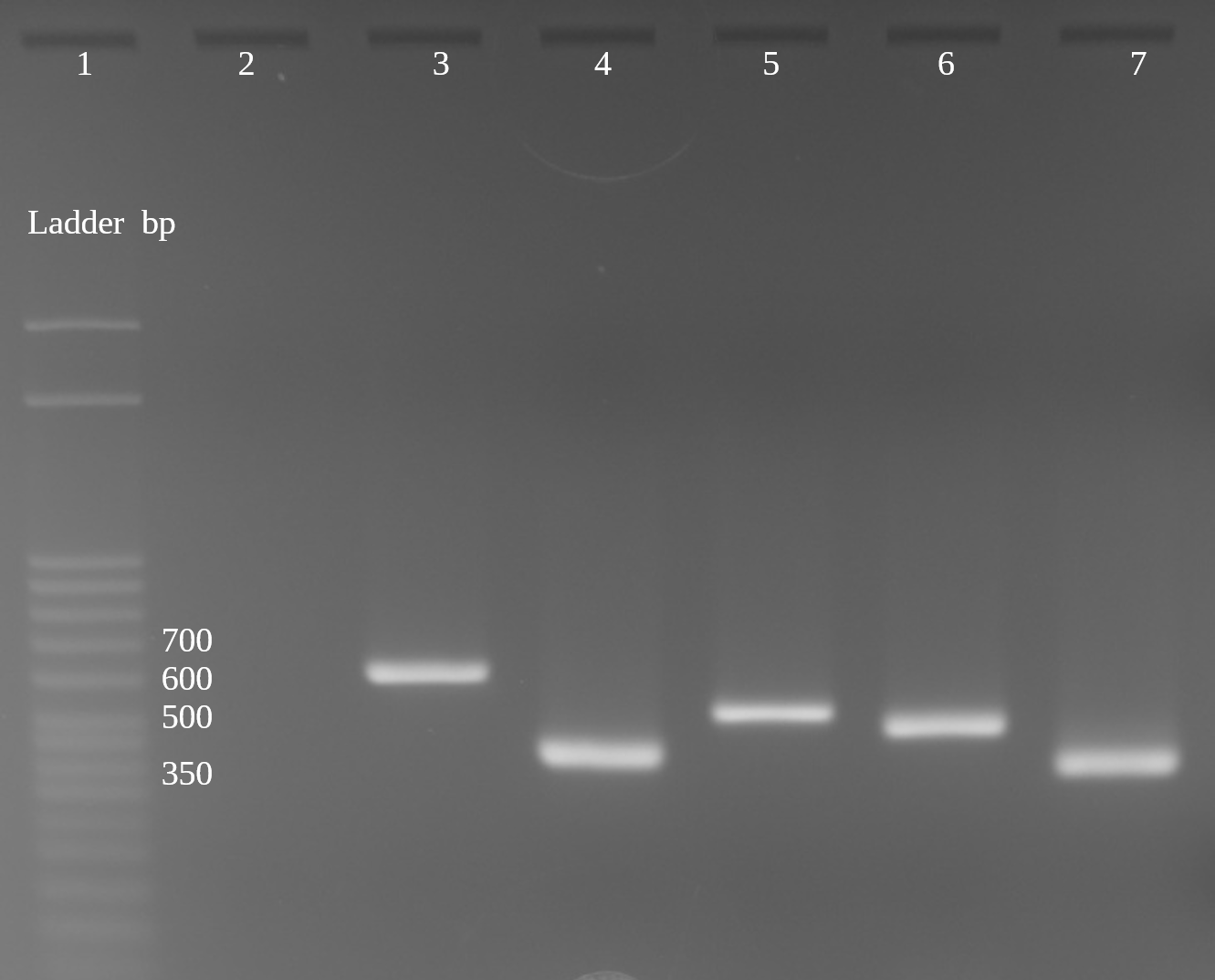

Supplement: S1 Fig — From left to right, lane 1 displays 50 bp DNA Step Ladder (Sigma), lane 2 is negative control, lane 3 displays first step PCR product (607 bp) and lines 6, 5, 4, and 7 display second step PCR products, respectively: ST1 (433 bp), ST2 (459 bp), ST3 (427 bp) and ST4 (399 bp). (TIF) [file pone.0210171.s001.tif]
